# Supplementary material for: Extending the Toolkit for Beauty: Differential Co-Expression of DROOPING LEAF-like and Class B MADS-box Genes during Phalaenopsis Flower Development
Source: Int J Mol Sci. 2021 Jun 29;22(13):7025. doi: 10.3390/ijms22137025 (PMC8268020; doi:10.3390/ijms22137025)
Supplement: Supplementary file 1 [file ijms-22-07025-s001.zip › Supplementary_Material.docx]

Supplementary Material

Supplementary Table S1. Conserved motifs of the putative promoters of the *PeDL2* and *DcDL2* genes found within the putative promoter of the *DcDL1* gene. The MEME motif numbers correspond to those of Figure 9. Only matches with motifs 2, 3, 4 and 9 were found, all with high false discovery rate (FDR).

| **MEME motif** | **Sequence** | **Target** | **Score** | ***p*-value** | **FDR** |
| --- | --- | --- | --- | --- | --- |
| **2** | CCGCCGG | DcDL1_P | 14.297 | 2.96e-05 | 0.177 |
| **4** | GYGGTGGRGAAGAWGACG | DcDL1_P | 5.66061 | 4e-05 | 0.238 |
| **9** | CTCCTCC | DcDL1_P | 13.7394 | 4.39e-05 | 0.131 |
| **9** | CTCCTCC | DcDL1_P | 13.7394 | 4.39e-05 | 0.131 |
| **3** | GTAASTGGTGGTACGG | DcDL1_P | 8.02424 | 5e-05 | 0.298 |
| **4** | GYGGTGGRGAAGAWGACG | DcDL1_P | 4.69091 | 9.75e-05 | 0.291 |

**Supplementary Table S2**. List of the primer sequences used. qPCR, quantitative real time PCR; Y2H, Yeast Two-Hybrid analysis; GAL4 DNA-binding domain (pGBKT7 vector); AD, GAL4 activation domain (pGADT7 vector).

| **Gene** | **Forward (5’-3’)** | **Reverse (5’-3’)** | **Use** |
| --- | --- | --- | --- |
| PeDL1 | AAGTCCTACTCCTTCATTGCTTACA | AGAAAAACAACATAAGCTGCTCGTT | PCR |
| PeDL2 | ACTCTCCACCATCTTCCTCC | CCCATGCATGCTATATCAAATGT | PCR |
| PeDL1_1 | GTGTGGGCACTGCAATCATCT | TCTTCTCTGGAGGTTTGACAAC | qPCR |
| PeDL1_2 | GGAAACAGGACTGGTTGTGTGA | TCTTCTCTGGAGGTTTGACAAC | qPCR |
| PeDL2_1 | ACTCTCCACCATCTTCCTCC | ATGGCCTCTGAAGCAGCGTTTGT | qPCR |
| PeDL2_2 | AAGTTGGCTGTGAAATGCATGAT | ATGGCCTCTGAAGCAGCGTTTGT | qPCR |
| PeMADS2 | GGGAAACTTACCGCGCTCTA | GATTGGGCTGATTCGGATGA | qPCR |
| PeMADS3 | ATTTCGTACCCAACCAAGCC | TGCAGTGCTAGACCCTACTT | qPCR |
| PeMADS4 | GATCTTCGCCTCGCTTGATA | ACCACAGAATCACACATAGCA | qPCR |
| PeMADS5 | GGAAGGGTTGGGCGTTAAAG | GTTCATGCGTTAGGGCTCTG | qPCR |
| 18S | TTAGGCCACGGAAGTTTGAG | ACACTTCACCGGACCATTCAA | qPCR |
| Actin | GGTATTGTTCTTGATTCTGGTGATGGTGTCA | GTCTTGGCAGTTTCCAACTCTTGCTCATAATC | qPCR |
| EF 1α | TGTGAAGAAGAAATGAAGTG | AACAACAGACTCAAAGACCT | qPCR |
| PeMADS2_AD | CCGGAATTCCGGATGGGGAGGGGGAAGATAGAGA | AGAGCTCAATTATGCAAGGCTAAGATCATGTG | Y2H |
| PeMADS2_BD | CCGGAATTCCGGATGGGGAGGGGGAAGATAGAGA | TCCGTCGACTTATGCAAGGCTAAGATCATGTG | Y2H |
| PeMADS3_AD | CCGGAATTCCGGATGGGGAGGGGGAAGATCGAGA | AGAGCTCAATCAGGCGAGACGTAGATCATG | Y2H |
| PeMADS3_BD | CCGGAATTCCGGATGGGGAGGGGGAAGATCGAGA | TCCGTCGACTCAGGCGAGACGTAGATCATG | Y2H |
| PeMADS4_AD | CCGGAATTCCGGATGGGGAGGGGGAAGATAGAGA | AGAGCTCAATCACGATCTTCGCCTCGCTTGA | Y2H |
| PeMADS4_BD | CCGGAATTCCGGATGGGGAGGGGGAAGATAGAGA | TCCGTCGACTCACGATCTTCGCCTCGCTTGA | Y2H |
| PeMADS5_AD | CCGGAATTCCGGATGGGGAGAGGGAAGATAGAGA | AGAGCTCAATCAATCAAAGCCAAACTCATGAC | Y2H |
| PeMADS5_BD | CCGGAATTCCGGATGGGGAGAGGGAAGATAGAGA | TCCGTCGACTCAATCAAAGCCAAACTCATGAC | Y2H |
| PeMADS6_AD | CCGGAATTCCGGATGGGTCGGGGAAAGATAGAGA | AGAGCTCAATTACTTATTTCCCTGCA | Y2H |
| PeMADS6_BD | CCGGAATTCCGGATGGGTCGGGGAAAGATAGAGA | TCCGTCGACTTACTTATTTCCCTGCA | Y2H |
| PeDL1_1_AD/BD | CCGGAATTCCGGATGGATCTGGTTTCTTCAAGGGA | AAGTCGACTTAGTTCTGGCGATCCATTTGTTT | Y2H |
| PeDL1_2_AD/BD | CCGGAATTCCGGATGCAGGATCCATGTAATGATTGT | AAGTCGACTTAGTTCTGGCGATCCATTTGTTT | Y2H |
| PeDL2_1_AD/BD | CCGGAATTCCGGATGGACTATGGCTCTCCTTCAGAC | AGGATCCAATTATTCCATGCGACGCTGTTGTC | Y2H |
| PeDL2_2_AD/BD | AGGATCCAAATGACGGACACAGTGACAGTTAAG | AAGTCGACTTATTCCATGCGACGCTGTTGTC | Y2H |


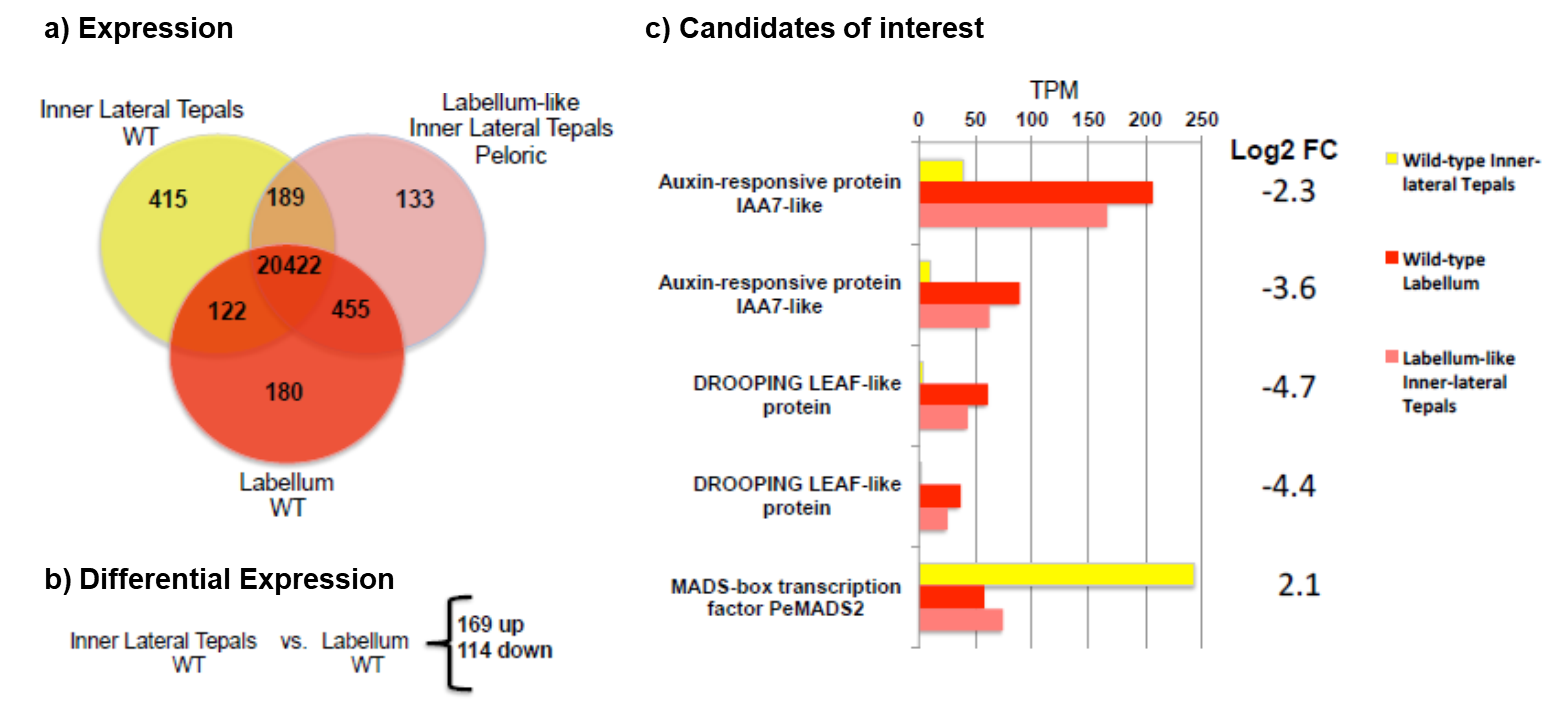


**Supplementary Figure S1.** Summary of RNA-seq analysis of *Phalaenopsis hyb*. “Athens”. a) Transcripts expressed in perianth organs of wild-type (WT) and peloric *Phalaenopsis* hyb. “Athens” with at least 1 TPM. The number of transcripts shared and specific for each floral organ are indicated within the circles. b) Number of differentially expressed genes between WT lateral inner tepals and labellum. c) Candidate transcripts of interest differentially expressed between lateral inner tepals of and labellum of WT *Phalaenopsis* hyb. “Athens”.


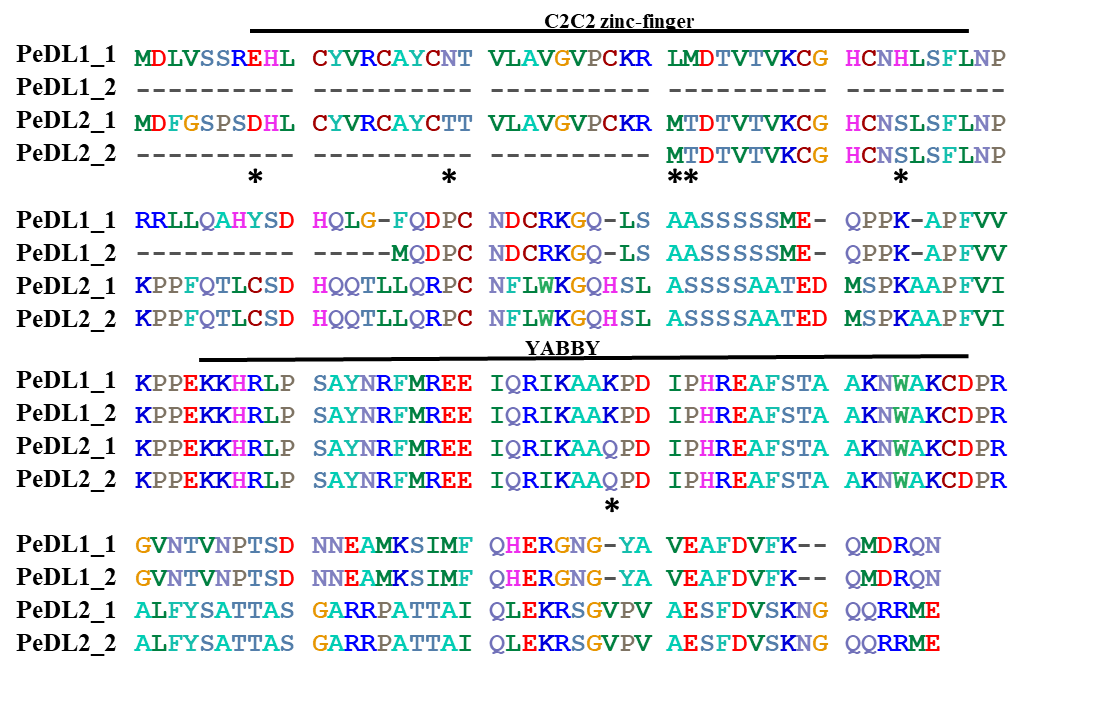


**Supplementary Figure S2.** Amino acid alignment of the different isoforms of the PeDL1 and PeDL2 proteins of *P. equestris*. The C2C2 zinc-finger and the YABBY domains are underlined. The asterisks indicate the variable residues within the C2C2 and YABBY domains of PeDL1_1 and PeDL2_1.


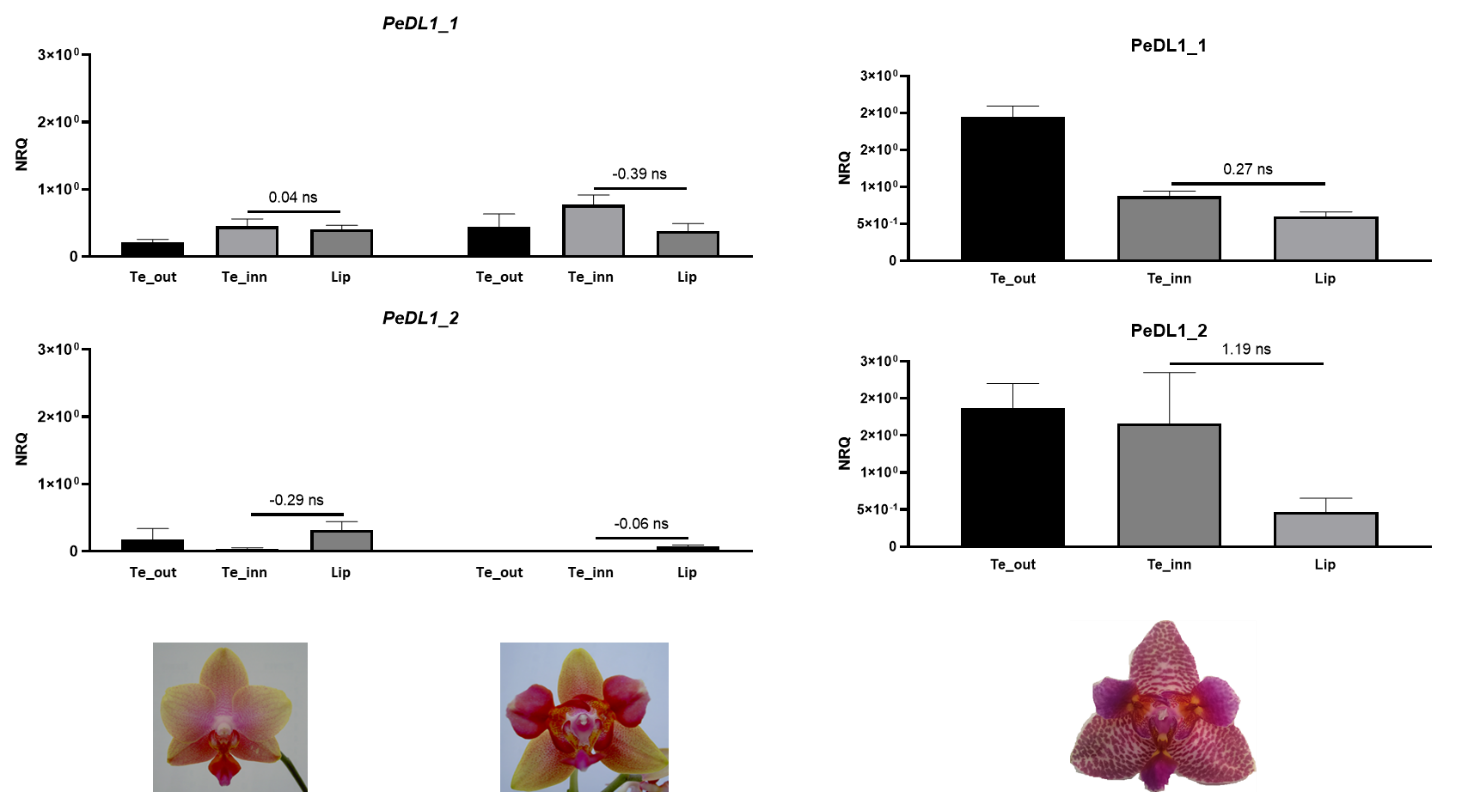


**(a)**

**(b)**

**(c)**

**Supplementary Figure S3.** Relative expression of the isoforms *PeDL1_1* and *PeDL1_2* in the perianth of the wild type **(a)** and peloric **(b)** *Phalaenopsis* hyb. “Athens” and of the peloric *Phalaenopsis* hyb. “Joy Fairy Tale” **(c)** at the B2 developmental stage (bud size 1-1.5 cm). The expression is reported as normalized relative quantity (NRQ). The vertical bars represent the SEM of the biological and technical replicates. The numbers above the horizontal lines are the mean differences of the expression between lateral inner tepals and labellum (Te_inn - Lip). ns, not significant. Te_out, outer tepals; Te_inn, lateral inner tepals or labellum-like structures that substitute the lateral inner tepals in the peloric mutant.

**
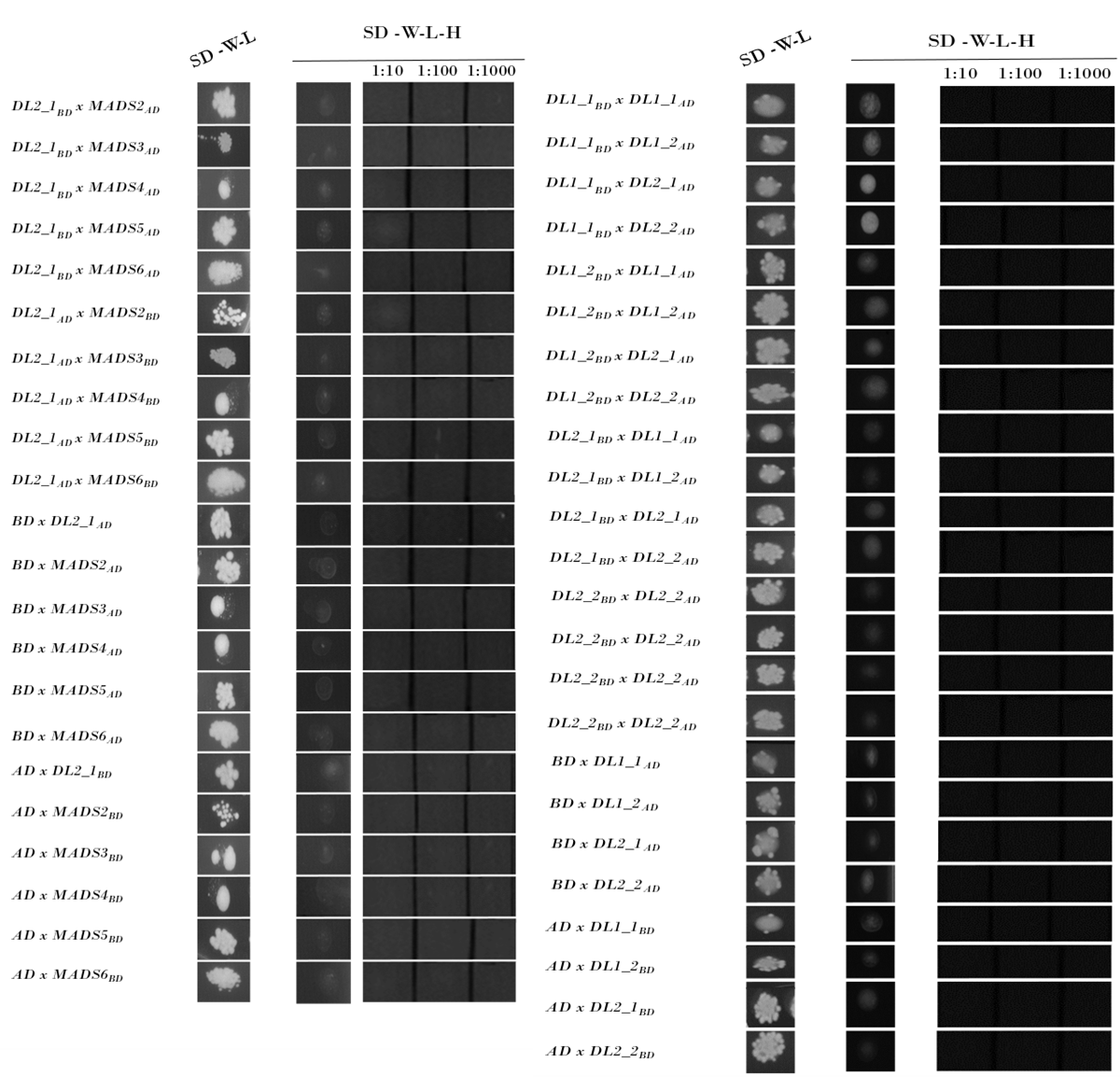
Supplementary Figure S4.** Interactions of the PeDL2_1 and PeMADS2-PeMADS6 (left) and of the different isoforms of PeDL1/2 (right) of *Phalaenopsis* in Yeast Two Hybrid analysis. After double transformations, yeast growth in absence of tryptophan and leucine (SD -W-L) indicates the plasmid presence; yeast growth in medium lacking tryptophan, leucine and histidine (SD -W-L-H) would indicate interaction between the two tested proteins. Double transformations conducted using one of the vectors empty are negative controls. 1:10, 1:100 and 1:1000 are the dilution factor applied to the yeast inoculate. BD, GAL4 DNA-binding domain (pGBKT7 vector); AD, GAL4 activation domain (pGADT7 vector).


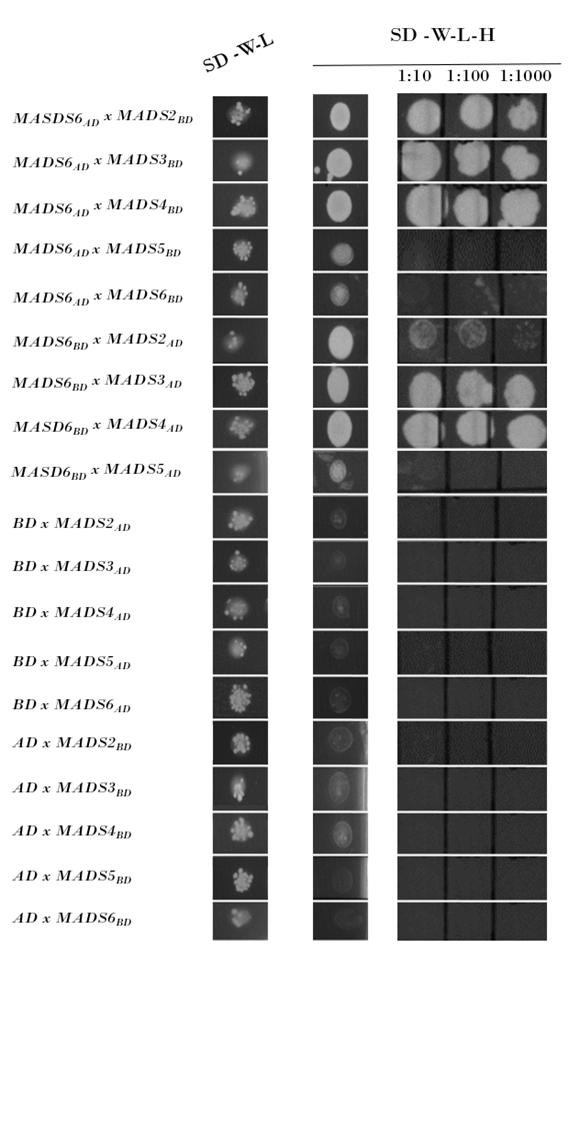


**Supplementary Figure S5.** Interactions of PeMADS2-PeMADS5 and PeMAD6 of *Phalaenopsis* in Yeast Two Hybrid analysis. After double transformations, yeast growth in absence of tryptophan and leucine (SD -W-L) indicates the plasmid presence; yeast growth in medium lacking tryptophan, leucine and histidine (SD -W-L-H) would indicate interaction between the two tested proteins. Double transformations conducted using one of the vectors empty are negative controls. 1:10, 1:100 and 1:1000 are the dilution factor applied to the yeast inoculate. BD, GAL4 DNA-binding domain (pGBKT7 vector); AD, GAL4 activation domain (pGADT7 vector).


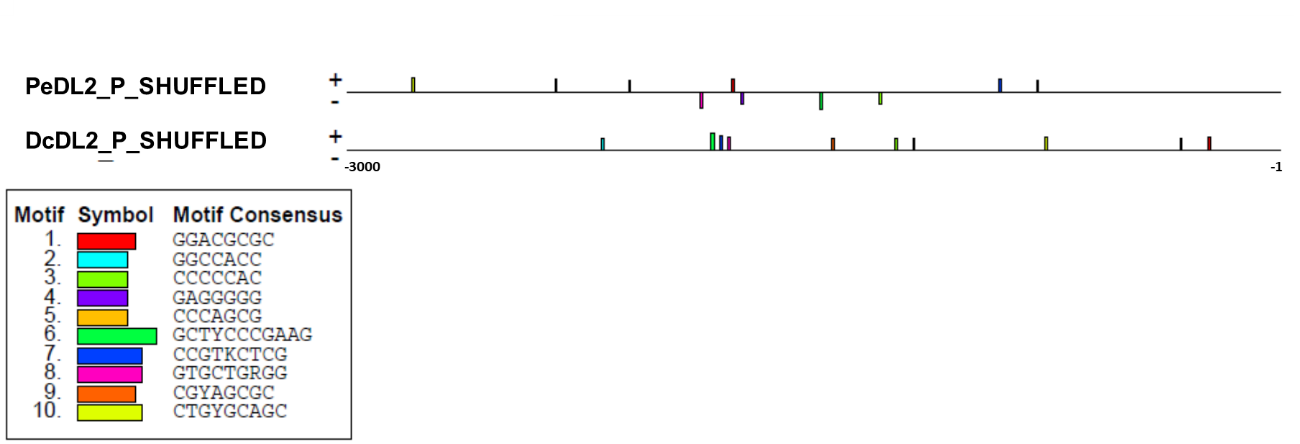


**Supplementary Figure S6.** Conserved motifs within the shuffled sequences of the putative promoters of the *DL2* genes of *P. equestris* and *D. catenatum*. Note that these motifs do not correspond to those found within the primary (not-shuffled) sequences (Figure 9). PeDL2_P_SHUFFLED and DcDL2_P_SHUFFLED are the shuffled nucleotide sequences spanning 3000 bp upstream the ATG translation start site of the *PeDL2* and *DcDL2* genes, numbered from -1 to -3000.


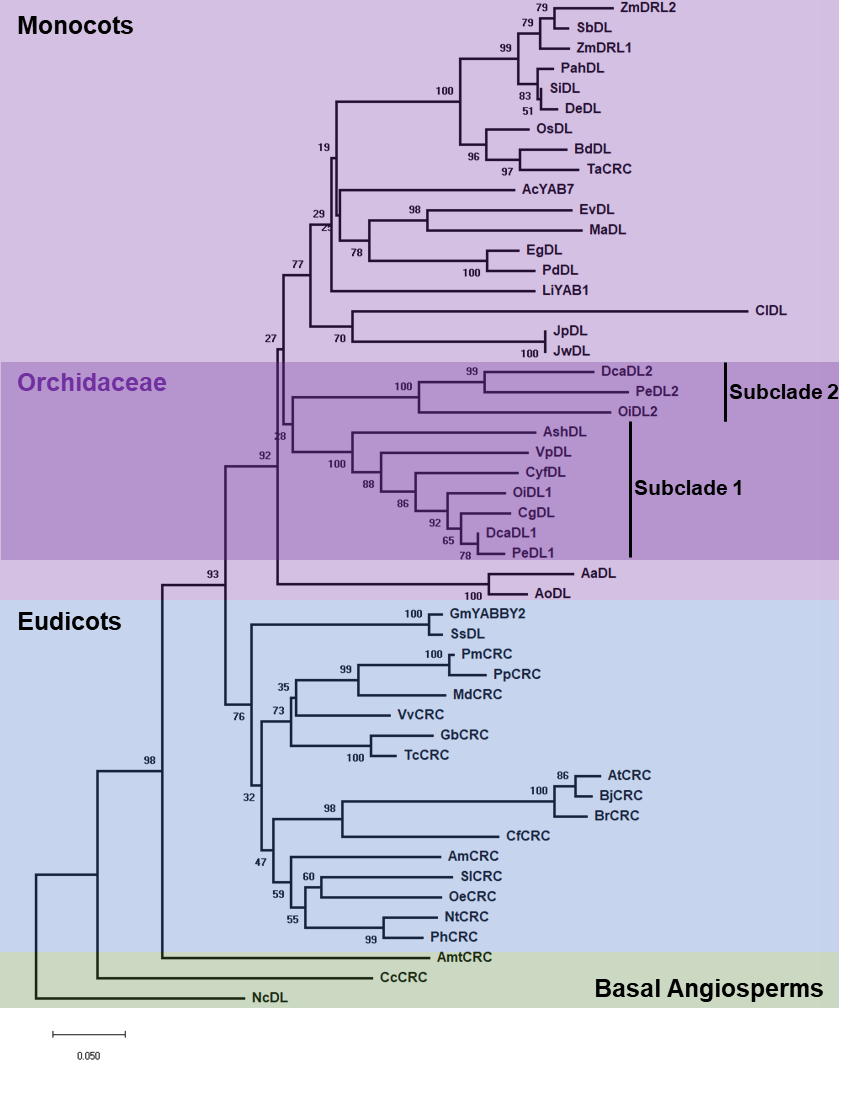


**Supplementary Figure S7.** Neighbor Joining tree of the CRC/DL proteins. The tree was constructed on the CRC/DL amino acid alignment of selected monocots and dicots (mainly from Chen et al., 2020 [42]) using MEGA software. The sequences were aligned using Clustal Omega. The numbers above the branches represent the bootstrap percentages (500 replicates). **Monocots**: AcYAB7 (*Ananas comosus*, XP_020105063), AshDL (*Apostasia shenzhenica*, PKA49723), AaDL (*Asparagus asparagoides*, BAI68347), AoDL (*Asparagus officinalis*, XP_020276732), BdDL (*Brachypodium distachyon*, KQK22884), ClDL (*Carex littledalei*, KAF3326740), CgDL (*Cymbidium goeringii*, ADI58463), CyfDL (*Cypripedium formosanum*, CFTC009810), DcDL1 and DcDL2 (*Dendrobium catenatum*, PKU69929 and PKU86051, respectively), DeDL (*Digitaria exilis*, CAB3496665), EgDL (*Elaeis guineensis*, XP_010939213), EvDL (*Ensete ventricosum*, RRT32785), JpDL (*Juncus prismatocarpus*, BAJ14106), JwDL (*Juncus wallichianus*, BAJ14110), LiYAB1 (*Lilium longiflorum*, ABP35569), MaDL (*Musa acuminata*, XP_009395243), OiDL1 and OiDL2 (*Orchis italica*, OITC006016 and comp7559, respectively), OsDL (*Oryza sativa*, AY494713), PahDL (*Panicum hallii*, XP_025797474), PeDL1 and PeDL2 (*Phalaenopsis equestris*, MW574592 and MW574594, respectively), PdDL (*Phoenix dactylifera*, XP_008785629), SiDL (*Setaria italica*, XP_012704688), SbDL (*Sorghum bicolor*, XP_021307124), TaCRC (*Triticum aestivum*, AAQ11881), VpDL (*Vanilla planifolia*, VPTC001074), ZmDRL1 and ZmDRL2 (*Zea mays*, GRMZM2G088309 and GRMZM2G102218, respectively). **Eudicots**: AmCRC (*Antirrhinum majus*, AAS10180), AtCRC (*Arabidopsis thaliana*, NP_177078.1), BjCRC (*Brassica juncea*, AAZ23116.1), BrCRC (*Brassica rapa*, XP_009105464), CfCRC (*Cynophalla flexuosa*, AAW83045), GmYABBY2 (*Glycine max*, XP_003517857), GbCRC (*Gossypium barbadense*, KAB2041458), MdCRC (*Malus domestica*, XP_008339784), NtCRC (*Nicotiana tobacum*, AAW83046), OeCRC (*Olea europaea*, XP_022846429), PhCRC (*Petunia x hybrid*, AAW83048), PmCRC (*Prunus mume*, XP_008243820), PpCRC (*Prunus persica*, XP_007223999.2), SlCRC (*Solanum lycopersicum*, XP_004239032.1), SsDL (*Spatholobus suberectus*, TKY69532), TcCRC (*Theobroma cacao*, EOY01637), VvCRC (*Vitis vinifera*, XP_010650015). **Basal Angiosperms**: AmtCRC (*Amborella trichopoda*, CAI47004.1), CcCRC (*Cabomba caroliniana*, BAJ83622), NcDL (*Nymphaea colorata*, XP_031490637).
